# PI3K/Akt Signal Pathway Involved in the Cognitive Impairment Caused by Chronic Cerebral Hypoperfusion in Rats

**DOI:** 10.1371/journal.pone.0081901

**Published:** 2013-12-10

**Authors:** Yi Shu, Hong Zhang, Tao Kang, Jun-jian Zhang, Ying Yang, Hui Liu, Lei Zhang

**Affiliations:** Department of Neurology, Zhongnan Hospital of Wuhan University, Wuhan, China; Imperial College London, Chelsea & Westminster Hospital, United Kingdom

## Abstract

Chronic cerebral hypoperfusion (CCH) is a common pathophysiological state that usually occurs in conditions such as vascular dementia and Alzheimer's disease, both of which are characterized by cognitive impairment. In previous studies we found that learning capacity and memory were gradually impaired with CCH, which altered the expression of synaptophysin, microtubule associated protein-2, growth associated protein-43, brain-derived neurotrophic factor, nerve growth factor, N-methyl-D-aspartate receptor subunit 1, cAMP response element-binding protein and tau hyperphosphorylation in the hippocampus. However, the molecular basis of cognitive impairment in CCH remains obscure. Here we explore the hypothesis that the phosphoinositide 3-kinase (PI3K)/protein kinase B (Akt) signal pathway is involved in this type of cognitive impairment. In order to determine if the expression of PI3K, Akt and phosphorylated Akt (p-Akt) proteins are altered at different stages of CCH with differing levels of cognitive impairment. we performed permanent, bilateral occlusion of the common carotid arteries (2-VO) to induce CCH. Adult male SD rats were randomly divided into sham-operated group, 2-VO 1 week group, 2-VO 4 weeks group and 2-VO 8 weeks group. Behavior tests were utilized to assess cognitive abilities, while western blots were utilized to evaluate protein expression. Rats in the 2-VO groups spent less time exploring novel objects than those in the sham-operated group, and the discrimination ratio of the 2-VO 8 weeks group and the sham-operated group were higher than chance (0.50). Escape latencies in the Morris water maze task in the 2-VO 1 week group were longer than those in the sham-operated group on day 4 and day 5, while escape latencies in the 2-VO 4 weeks group were longer than those in the sham-operated group from day 3 to day 5. Escape latencies in 2-VO 8 weeks group were longer than those in the sham-operated group from day 2 to day 5. NE (northeast) square swimming times in the 2-VO 1 week group, 2-VO 4 weeks group and 2-VO 8 weeks group were shorter than that in the sham-operated group. Western blotting showed that the PI3K expression in the 2-VO 1 week group was lower than that in sham-operated group, while p-Akt expression in the 2-VO 8 weeks group was higher than that in the sham-operated group. There was a linear relationship between the PI3K expression and the discrimination ratio, as well as a linear relationship between the PI3K and NE square swimming time. Thus, we propose that the PI3K/Akt signal pathway is an important cell pathway that is associated with the cognitive impairment following CCH.

## Introduction

Alzheimer's disease and vascular dementia are the most common causes of cognitive impairment in the elderly. During the last decade, a number of clinical studies have identified cerebral hypoperfusion in brain regions associated with the cognitive functions both in Alzheimer's disease and vascular dementia patients[1]–[3]. An increasing number of experimental studies also support the notion that a reduced blood supply to brain is a decisive factor in the pathogenesis of the cognitive impairment. Despite this, the underlying molecular pathways that lead to the cognitive impairments are poorly defined.

Permanent bilateral occlusion of the common carotid arteries (2-vessel occlusion, 2-VO) is the most commonly used rat model to investigate the mechanisms of CCH-induced cognitive impairment and evaluate the therapeutic efficacy of drugs used to treat CCH-related cognitive impairment [4], [5]. Using the 2-VO model, our previous studies found that after 2-VO treatment, the learning capacity and memory of the subjects are gradually impaired and the expression of synaptophysin, microtubule associated protein-2, growth associated protein-43, brain-derived neurotrophic factor, nerve growth factor, *N*-methyl-D-aspartate receptor subunit 1, phosphorylated cyclic AMP-responsive element binding protein and tau hyperphosphorylation are altered within the hippocampus. Either pretreating the CCH groups with Angelica (a traditional Chinese herb), or providing an enriched environment significantly reverses the learning and memory impairments and the expressions of these proteins [6]–[12]. The underlying mechanisms connecting CCH-induced protein expression and the observed behavioral impairment remains largely unknown.

The phosphoinositide 3-kinase (PI3K)/protein kinase B (Akt) pathway is widely expressed during central nervous system development. In neurons, PI3K is induced by growth factors such as nerve growth factor, brain-derived neurotrophic factor, glial cell line-derived neurotrophic factor, neurotrophin-3, insulin-like growth factor-1 and insulin, as well as extracellular matrix proteins, cytokines and neurotransmitters. This pathway seems to be particularly important for mediating neuronal survival under a wide variety of circumstances [13]. Akt, plays a critical role in controlling survival and apoptosis, and is activated by growth factors to function in a pathway involving PI3K kinase [14]. The PI3K/Akt pathway can deactivate pro-apoptotic mediators, and activate anti-apoptotic proteins [15]. It is well known that the PI3K/Akt signaling pathway can mediate cell survival, differentiation and metabolism, which participate in neurocyte nutrition, angiogenesis, along with learning and memory [16]–[19]. It is also involved in the pathogenesis of acute cerebrovascular disease, neurodegeneration diseases, epilepsies and other neurological disorders [20]–[24]. Currently, there are few reports regarding the role that the PI3K/Akt pathway plays in the cognitive impairment caused by CCH. In this study, we test the dynamic changes of the PI3K/Akt proteins in the hippocampus after the 2-VO induced CCH and clarify the correlation between these proteins levels and the cognitive impairment.

## Materials and Methods

### Animal's Model

Adult male, specific pathogen free Sprague-Dawley (SD) rats (aged 8–10 weeks and weighing 230–250 g) were acquired from the Animal Research Center of Wuhan University. All animals were housed two to three per cage at a temperature of 22–24 degree C with a regular 12 hour light-dark cycle and free access to water and food. This study was carried out in strict accordance with the recommendations in the Guide for the Care and Use of Laboratory Animals of the National Institutes of Health. The protocol was approved by the Committee on the Ethics of Animal Experiments of the Medical School of Wuhan University. Twenty-four rats were randomly divided into a sham-operated group (n = 6) and a 2-VO operation group (2-VO group, n = 18). The 2-VO operation group was divided into three subgroups:a one week of 2-VO group (2-VO 1 week, n = 6), a four weeks of 2-VO group (2-VO 4 weeks, n = 6) and an eight weeks of 2-VO group (2-VO 8 weeks, n = 6).

Permanent bilateral occlusion of the common carotid arteries (2-VO) was used to induce CCH as described previously [6]–[12]. Before surgery the rats were not supplied food for 12 h and water for 4 h. Rats were anesthetized with 10% chloral hydrate(350 mg/kg, i.p.)and were allowed to breathe spontaneously throughout the surgical procedure. Both common carotid arteries were exposed via a midline cervical incision and were double-ligated with silk sutures. The sham-operated animals were treated in a similar manner, except that the common carotid arteries were not occluded. During the surgery, body temperature was maintained at 37–38 degree C. Following surgery, rats were placed on a homeothermic tapetum until they recovered from the anesthesia and then placed in clean and ventilated cages and provided with food and water.

### Object Recognition Test

Rats have a tendency to interact more with a novel object than with a familiar (sample) object, which can be used for testing rats' non-spatial memory performances. Following previous protocol [11], [25], the three 2-VO groups were respectively tested after 1, 4, and 8 weeks following CCH surgery. The sham-operated group started the object recognition task 8 weeks after receiving surgery. Before the experiments, the rats were handled daily for 2 min per day for 3 days. Initially, the animals were carried into the testing room in their home cages, and the objects were placed in the test box (50 cm long ×38 cm wide ×35 cm high). Observations began when the rats were placed in the apparatus for the object recognition test. Each animal received a total of two trials for a total duration of 15 minutes (min). The first trial (10 min) was the sample trial in which two identical objects were presented in the left and right corners of the test box. The second trial (5 min) was the test trial and was conducted in the same manner as the first trial, except that a new object replaced one of the sample objects. The inter-trial interval between the sample trial and test trial was 1 h. When each trial was over, the animals were returned to their home cages. After each trial, the objects, walls and floor of the test box were cleaned with 70% isopropyl alcohol and allowed to dry prior to starting the next trial. A video camera mounted on the ceiling above the test box recorded the animal's behavior. The objects presented were similar in both their materials and size, but differed in color and shape (e.g. porcelain cups, glass bottles, and glass ashtrays). The objects were tested on animals in a pilot study to ensure that the animals did not exhibit behaviors toward the objects that indicated they had any aversive or salient value. Exploration was defined as an animal directing its nose towards the object at a distance of less than 2 cm and/or touching the object with its nose. Turning around, rearing up onto, and sitting on the object were not considered exploration. The time spent exploring each object was measured with a stop watch. This common behavior exhibited by many mammals is known as familiarity discrimination. Statistical analysis of object exploration time and the discrimination ratio [T_N_/(T_N_+T_F_), T_N_ =  time spent exploring the novel object; T_F_ =  time spent exploring sample object] was performed with repeated measures and one- or two-way ANOVA followed by a Bonferroni multiple group comparison. A one sample t-test was used to determine whether the discrimination ratio was significantly different from chance (50%).

### Morris Water Maze Test

Spatial learning and memory performances were evaluated using the Morris water maze (MWM) tests [6]–[12]. The maze consisted of a circular and galvanized steel pool (120 cm in diameter and 60 cm in depth) that was filled to a depth of 32 cm with water at 22–26 degree C. The water was rendered opaque by the addition of a non-toxic, water soluble dye. A hidden circular platform (9 cm in diameter) was submerged approximately 2 cm below the surface of the opaque water and was kept in the northeast (NE) quadrant at the same location throughout the training period. The maze was virtually divided into four equally-spaced quadrants delineated by the cardinal points north, east, south, and west. The maze, located in a large and quiet test room, was surrounded by many visual cues (e.g. the experimenter, ceiling lights, rack, pictures), which were visible from within the pool and could be used by the rats to learn the location of the hidden platform. Locations of the cues were unchanged throughout the experiment. The experimenter did not change his location throughout the duration of the trials, since he was also a visual cue. A closed-circuit television camera was mounted onto the ceiling directly above the center of the pool and recorded swimming trajectories and parameters to an electronic image analyzer (Institute of Materia Medica, Chinese Academy of Medical Sciences, Beijing, China).

All rats (including the sham-operated group) were subjected to daily MWM tests after completing the object recognition test. Rats received four trails per day for five consecutive days with a constant interval of 1 hour (h). Rats were gently placed in the water in one of four quadrants facing the wall of the pool. The starting quadrant was varied randomly over the trials, and rats were allowed 60 second (s) to find the hidden platform. The actual time to find the hidden platform was recorded if it was less than 60 s. If the time exceeded 60 s, the latency time was recorded as 60 s. Regardless of whether it found the platform, the rat was placed on the platform for 20 s. For all trials, the escape latency and distance traveled before reaching the platform were measured. A single 30 s probe trial without the platform was given immediately after the five-day training period, and the time spent in the target quadrant (NE) where the platform had been placed during training was recorded.

### Western Blotting

Rats of each group were anesthetized with 10% chloral hydrate (350 mg/kg, i.p.) and the brain was rapidly removed and dissected on the ice to obtain the hippocampus on the 1^st^ day after the MWM tests [6]–[12]. All the samples were frozen immediately in liquid nitrogen and stored at −80 degree C. The hippocampus was homogenized in RIPA buffer (Beyotime, China) supplemented with the protease inhibitor PMSF (100 ug/ml, Solarbio, China) on ice. The homogenate was centrifuged at 10,000×g for 10 min, and the supernatant was stored at −20 degree C. Total protein concentrations were determined with a modified BCA assay [6]–[12] (Beyotime, China). Equal amounts (20 ul) of protein from each sample (3 ug/ul) were mixed with 20 ul sample buffer and 60 ul RIPA buffer and then boiled for 5 min. Proteins were separated by electrophoresis on 10% polyacrylamide gels. Separated proteins were transferred onto PVDF membrane where PI3K was 300 mA for 2 h, Akt and p-Akt were 300 mA for 90 min at 4 degree C (PI3K 110 kDa, Akt 60 kDa, p-Akt 60 kDa). The membrane was blocked for 2 h at room temperature with 5% dried, defatted milk in TBS buffer (50 mM Tris-HCl, pH 7.5, 150 mM NaCl) containing 0.1% Tween-20 (TBST). Blots were probed with primary antibodies against rabbit monoclonal PI3K (1∶400, Cell Signaling Technology, USA), rabbit monoclonal Akt (1∶1000, Cell Signaling Technology, USA) and rabbit monoclonal p-Akt (1∶1000, Cell Signaling Technology, USA) for 2 h at room temperature. After washing with TBST, the membranes were incubated for 2 h at room temperature with a secondary anti-rabbit antibody conjugated to horseradish peroxidase (KPL, USA) and developed using the enhanced chemiluminescence system (GE Healthcare, Canada). Negative controls did not contain primary antibody. The relative optical densities of the specific bands visible on X-ray film were scanned and measured by image analysis software (BandScan 5.0). All relative intensities were normalized to β-actin expression.

### Statistical Analysis

All data were analyzed using Graph Pad Prism 5.01 software(GraphPad, San Diego, California, USA). Differences in exploring time for the sample or novel object in the object recognition and escape latency in the MWM tests were analyzed using two-way ANOVA followed by the Bonferroni multiple group comparison. Statistical analysis of other data was performed by one-way ANOVA followed by a post Turkey test. The comparison between the sample mean and general mean were performed by student's t-test. The correlation between the performance in behavior tests and the protein expression were calculated using Spearman linear correlation analysis. Data were expressed as mean ± standard deviation (SD).

## Results

### Object Recognition Test

**Figure 1:**
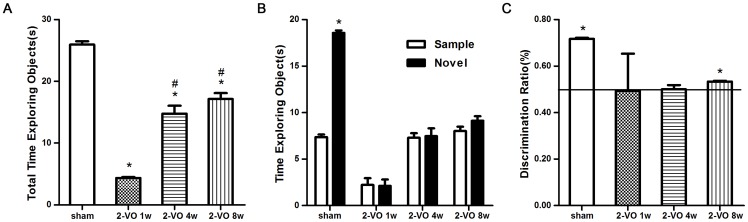
shows the effects of 2-VO treatment on the object recognition test. (A) Total time spent exploring the sample and the novel objects. (B) Time spent exploring the novel objects compare to the sample objects of rats in the object recognition test after 1 h inter-trial interval. (C) The discrimination ratio of rats in the test. Values are mean ±SD. In panel A, * *P*<0.01 vs. sham-operated group; # *P*<0.01 vs. 2-VO 1 week group. In panel B, * *P*<0.01 time spent in the novel object vs. the sample object in sham-operated group. In panel C, the horizontal line represents equal exploration of the novel and sample objects; * *P*<0.05 compared to the chance (50%). 2-VO: bilateral ligation of the common carotid arteries (2-vessel occlusion).

Rats undergoing the 2-VO procedure spent significantly less time exploring both the sample and novel objects relative to the sham-operated control [F (3,20) = 109.1, *P*<0.0001]. The total exploring time increased in the 2-VO 4 weeks group and the 2-VO 8 weeks group when compared with the 2-VO 1 week group (*P*<0.01), but there was no difference between the 2-VO 4 weeks group and the 2-VO 8 weeks group (*P*>0.05, see Figure 1A). Rats expressed similar motivation and spontaneous attraction for exploring the objects in the 2-VO 4 weeks and 8 weeks group, independent of the 2-VO surgery and environmental conditions; but in the 2-VO 1 week group the surgery itself made a critical differences in the task.

In the recognition trials, ANOVA revealed a significant effect of the 2-VO [F(3,40) = 124.53; *P*<0.0001], as well as an object ×2-VO interaction [F(3,40) = 46.65; *P*<0.0001]. The Bonferroni post-test indicated that the sham-operated rats explored the novel objects longer than the sample objects after 1 h inter-trial interval (*P*<0.01). The 2-VO group failed to explore the novel object more than the sample object after 1 h inter-trial interval. In addition, there was no significant difference within the two groups between the times spent on exploring the two objects (see Figure 1B).

For the discrimination ratio (Figure 1C), the 2-VO 1 week and 4 weeks group rats spent an equal amount of time exploring each object. The level of exploration of the novel object did not differ from chance (49.33±15.98% and 50.00±18.00%, *P*>0.05). In contrast, the 2-VO 8 weeks group and the sham-operated group spent more time exploring the novel object than the sample object, and the level of exploration of the novel object was significantly greater than chance (53.33±0.33% and 71.67±0.49%, *P*<0.05).

### Morris Water Maze Test

**Figure 2:**
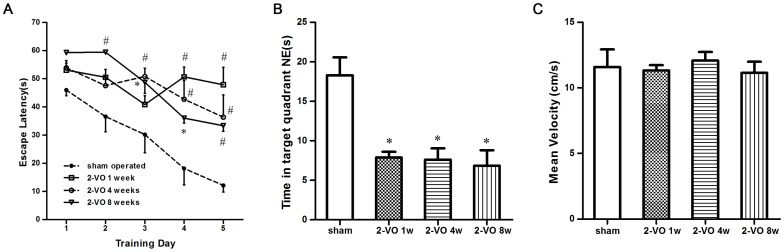
shows the effects of 2-VO treatment on the Morris water maze test. (A) Changes in escape latency during the training days. (B) Time spent in NE phase during the probe trials (swimming 60 s without platform). (C) Swimming speeds of escape latency. Values are mean ±SD. In panel A, # *P*<0.01, * *P*<0.05 vs. sham-operated group. In panel B, * *P*<0.01 vs. sham-operated group. 2-VO: bilateral ligation of the common carotid arteries (2-vessel occlusion).

Rats were subjected to 5 days of trials in the MWM tests to investigate their spatial learning ability after the object recognition test. Escape latencies decreased significantly across the five days of training [F (3, 100) = 25.86, *P*<0.0001], and there were significant differences between the four groups [F (4, 100) = 16.28, *P*<0.0001]. Training day interacted significantly with rat group [F (12, 100) = 2.52, P = 0.0062]. A Bonferroni post-hoc test indicated that the 2-VO 1 week group took longer to find the platform than the sham-operated group on both day 4 and day 5 (*P*<0.01). The 2-VO 4 weeks group spent more time on escape latency from day 3 to day 5 (*P*<0.01), and the 2-VO 8 weeks group spent a longer amount of time from day 2 to day 5 (*P*<0.05, or *P*<0.01). There were no significant differences in escape latency among the 2-VO 1 week, 4 weeks and 8 weeks group on days 4 and 5 (Figure 2A).

In the probe trials, memory was evaluated by measuring the time spent in the target quadrant without the platform (Figure 2B). We found a significant difference in the time spent in the target quadrant among the four groups [F (3, 23) = 10.28, *P* = 0.0003]. Turkey's post-hoc test indicated that the 2-VO group spent less time in this quadrant than the sham-operated group (*P*<0.01), but there was no difference between the 2-VO groups in swimming time in the NE phases (*P*>0.05). To determine whether the group differences in escape latency were due to differences in swimming ability, the swim speed was calculated for each group.We found that the 2-VO procedure did affect swimming rate [F (3, 20) = 0.2097, P = 0.8885; Figure 2C].

### Expressions of PI3K/Akt Pathway Proteins

**Figure 3:**
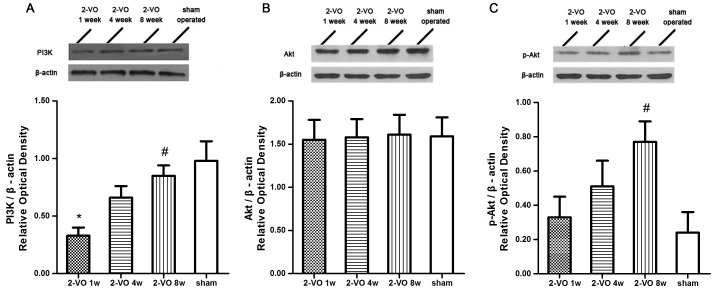
shows the effects of 2-VO treatment on the protein levels of PI3K (A), Akt (B) and p-Akt (C) by Western blotting. The upper panels show representative Western blotting results from hippocampus protein extracts probed with the corresponding antibodies. The protein bands in the upper panels were scanned and quantities, with the results being indicated as ROD (relative optical density). Values are mean ±SD. In panel A, * *P*<0.01 vs. sham-operated group, # *P*<0.05 vs. 2-VO 1 week group. In panel C, # *P*<0.05 vs. sham-operated group. 2-VO: bilateral ligation of the common carotid arteries (2-vessel occlusion).

The expressions of PI3K, Akt and p-Akt were assessed with Western blotting (see Figure 3A, 3B and 3C). There were a significant difference between the 2-VO treatment groups in PI3K and p-Akt expression [F(3, 20) = 6.148, *P* = 0.0039; F(3, 20) = 3.326, *P* = 0.0405]. The PI3K and p-Akt protein expression in the hippocampus was gradually up-regulated. Turkey's test indicated that the PI3K expression differed between the 2-VO 1 week group and the sham-operated group (*P*<0.01), and between the 2-VO 8 weeks group and 1 week group (*P*<0.05). A post-hoc Turkey's test indicated that the p-Akt is expressed at high levels in the 2-VO 8 weeks group than that in the sham-operated group (*P*<0.05). The Akt protein expression showed no differences between the groups [F(3, 20) = 0.0126,*P* = 0.9980].

### Correlation Analysis

**Figure 4:**
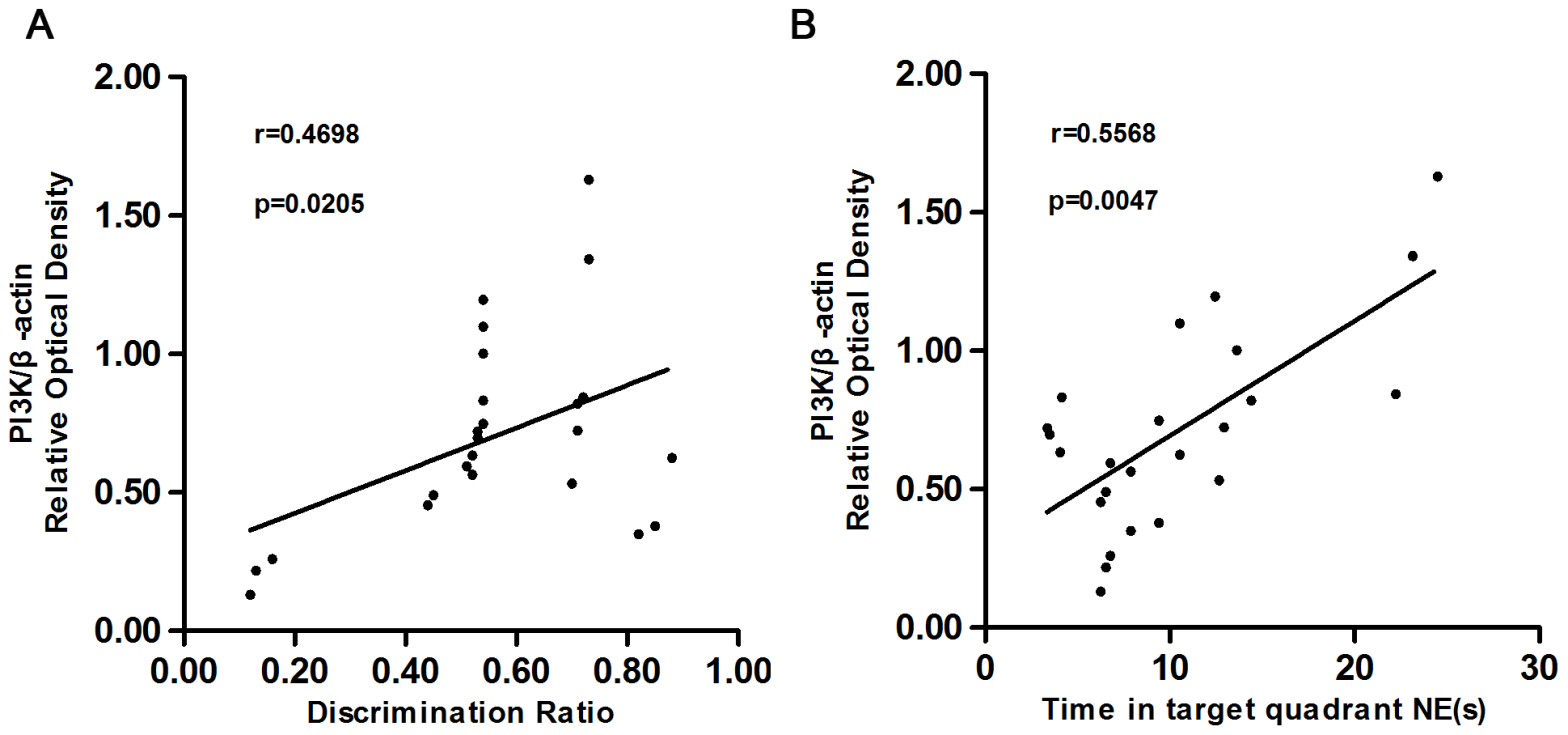
shows the scatter plots relating the discrimination ratio in the object recognition test (A), the swimming time spent in the phase NE (B), with the protein level of PI3K in the hippocampus among 2-VO groups and the sham-operated group. The change of the PI3K protein expression in the hippocampus treated with 2-VO was positively correlated with both the discrimination ratio (r = 0.4698, *P* = 0.0205), and the swimming time spent in the phase NE (r = 0.5568, *P* = 0.0047).

**Figure 5:**
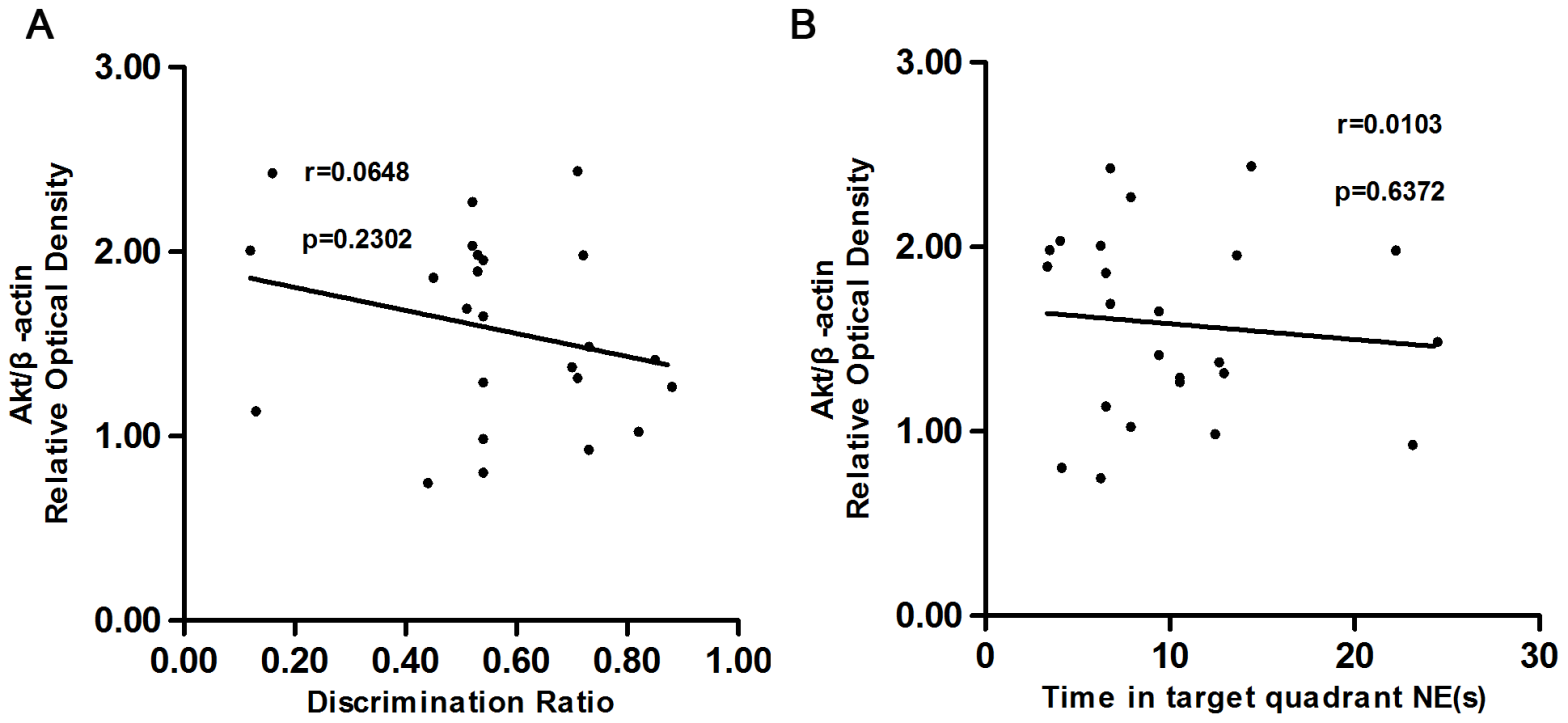
shows the scatter plots relating the discrimination ratio in the object recognition test (A), the swimming time spent in the phase NE (B), with the protein level of Akt in the hippocampus among 2-VO groups and the sham-operated group. There was no correlation between the Akt protein level expression with either the discrimination ratio (r = 0.0648, *P* = 0.2302), or the swimming time spent in the phase NE (r = 0.0103, *P* = 0.6372).

**Figure 6:**
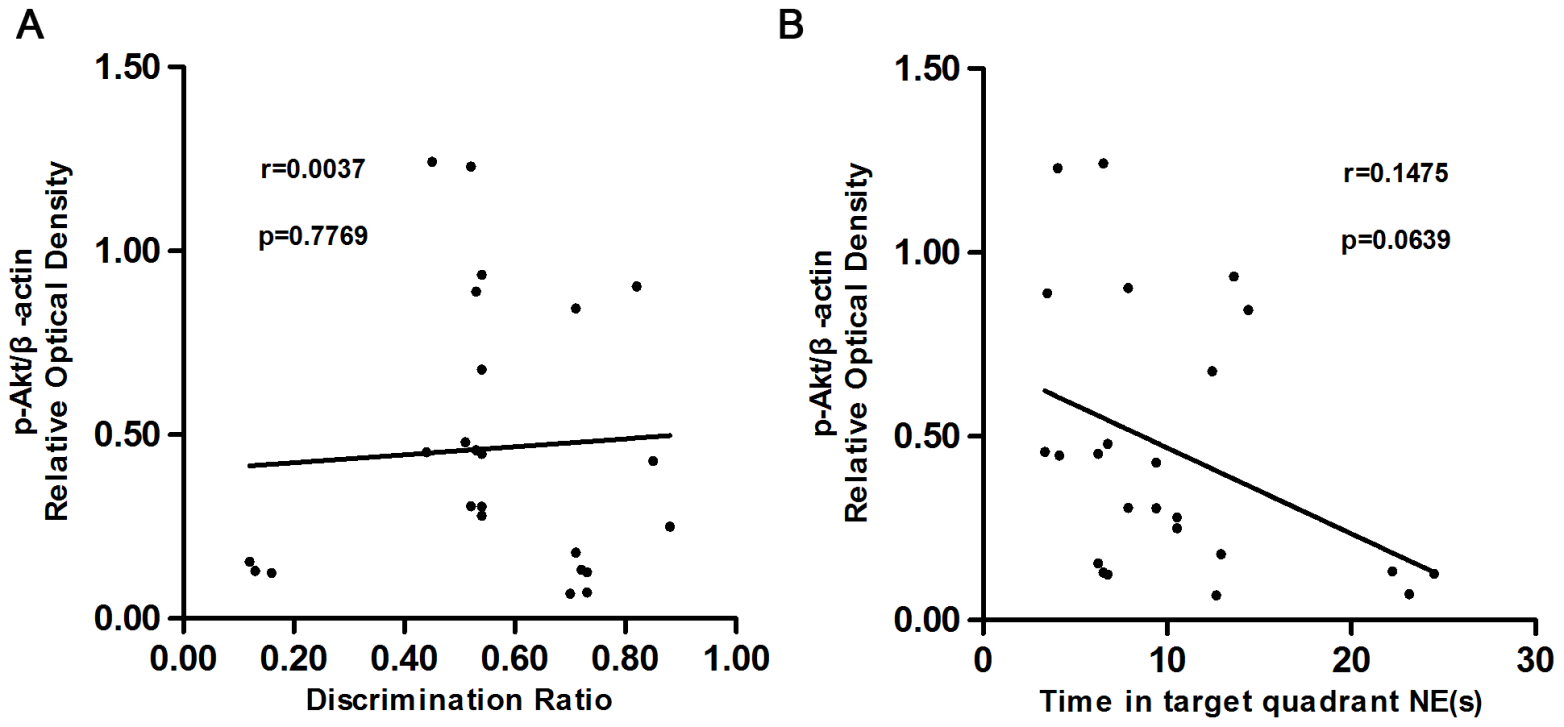
shows the scatter plots relating the discrimination ratio in the object recognition test (A), the swimming time spent in the phase NE (B), with the protein level of p-Akt in the hippocampus among 2-VO groups and the sham-operated group. There was no correlation between the p-Akt protein level expression with either the discrimination ratio (r = 0.0037, *P* = 0.7769), or the swimming time spent in the phase NE (r = 0.1475, *P* = 0.0639).

The linear relationship between the behavioral performance and the expression levels of PI3K, Akt and p-Akt proteins were also assessed. In the 2-VO groups, the PI3K protein expression in the hippocampus was positively correlated with both the object discrimination ratio (r = 0.4698, *P* = 0.0205), and the time spent swimming in the NE quadrant (r = 0.5568, *P* = 0.0047; see Figure 4A and 4B). There was no correlation between the Akt or p-Akt protein level expression with either the discrimination ratio or the time spent in the NE quadrant [see Figure 5A (r = 0.0648, *P* = 0.2302) and 5B (r = 0.0103, *P* = 0.6372); Figure 6A (r = 0.0037, *P* = 0.7769) and 6B (r = 0.1475, *P* = 0.0639).

## Discussion

Chronic cerebral hypoperfusion (CCH) is involved in the pathogenesis of the cognitive impairment [4]–[12]. For this reason, permanent bilateral occlusion of the common carotid arteries (2-VO) in rats has been utilized to investigate the effects of CCH on cognitive dysfunction and the neurodegenerative processes. With the help of the 2-VO model, the causal and sequential interactions of cerebral hypoperfusion, neuronal injury and memory deficits can be elucidated. Over the years, the 2-VO model has generated a large amount of data, revealing the 2-VO related pattern of cerebral hypoperfusion and metabolic changes, the learning and memory disturbances, failure of neuronal signaling, and the neuropathological changes in the hippocampus [26]. Although 2-VO is a widely accepted model with the characteristics of long-lasting cerebral blood flow (CBF) reduction and continuing cognitive impairment, a shortcoming of the 2-VO model is that CBF drops rapidly and suddenly in the early stage. That is, during the acute phase (from the start of occlusion to 2–3 days) of 2-VO, the CBF declines more rapidly than that found in patient groups with CBF reduction. CBF in the cortex and hippocampus was reported to drop to 35–45% and around 60% of the control, respectively, soon after surgery [27]–[29]. It has been suggested that CBF in 2-VO rats begins to recover at week 1 after surgery and almost returns to the baseline level at week 8 [28].

In this study, the cognitive performance was evaluated at weeks 1, 4 and 8 after CCH caused by the 2-VO model. To evaluate non-spatial memory, the object recognition test was used. This method measures a specific form of episodic memory in rats and mice [25], [30]. The novelty-preference paradigm is based on the natural motivation of rodents for novelty and their ability to remember previously encountered objects [31], which is closely related to conditions in which human recognition memory is measured. Under normal conditions, rats spend more time with a novel object than a familiar (sample) object. The object recognition test requires little training and induces less stress relative to the MWM tests [30].Only a few studies have directly examined the object recognition test following CCH [11], [32], [33]. In the current study, the results indicated that both the non-spatial and spatial cognitive functions were impaired after CCH caused by 2-VO model, especially at the early stages (2-VO 1 week). As the post-operative time increased, the non-spatial and spatial cognitive functions improved relative to animals tested shortly after occlusion. This observation is consistent with our previous studies and those of others [8], [11], [26], [34], [35]. Although the underlying mechanisms may be complicated, we proposed that this change may be associated with artery dilation, the recruitment of nonperfused capillaries and angiogenesis that causes blood flow to partly recover at 2-VO 8 weeks [6], [26].

The PI3K/Akt signaling pathway plays a central role in regulating cell growth, proliferation and survival under physiologic and pathologic conditions[36]. Gao et al [37] showed that ischemic postconditioning enhanced phosphorylation of Akt. The inhibition of Akt activity partly abolished the protective effects of postconditioning. In cultured hippocampal neurons, PI3K/Akt promotes neurite initiation, outgrowth, and the stability of growth cones [38]–[41], as well as the growth and branching of dendrites [42]. Many neuroprotectants, including propofol [43], humanin [44], estradiol [45], [46], melatonin [47], isoflurane [48], atorvastatin and tissue-type plasminogen activator [49], exert their protective effects through the PI3K/Akt pathway. An increasing number of studies suggest that insulin plays a role in the pathogenesis of Alzheimer's disease. Moreover several studies have reported an increased risk of dementia in subjects with non-insulin-dependent diabetes mellitus [50]. In order to test this hypothesis, Castri et al [51] analyzed the PI3K/Akt pathway following an in vitro challenge with insulin in peripheral blood mononuclear cells from patients with Alzheimer's disease compared with normal controls. Their major finding is that activation of the PI3K pathway was blunted in the peripheral cells from all of the patients with Alzheimer's disease, independent of the extent of cognitive decline. To further examine the mechanisms of therapeutic acetylcholinesterase inhibitors currently utilized in the treatment of Alzheimer's disease, Takada-Takatori et al [52] reported that the PI3K/Akt signaling pathway is involved in the neuroprotective effects of donepezil and galanthamine, but not that of tacrine. Furthermore, LY294002, a PI3K inhibitor, suppressed the neuroprotective effect of donepezil and galanthamine, but not that of tacrine. Using the vascular dementia model, Gong et al [53] reported that p-Akt but not Akt expression, were significantly decreased in the hippocampus at 1, 2 and 4 months following 2-VO injury. Unfortunately, many of these studies only used PI3K inhibitors or activators to study the role of PI3K, and never directly measured the amount of PI3K. In this study, we firstly demonstrated that the expression of PI3K, Akt and p-Akt proteins in the hippocampus after CCH for 1 week, 4 weeks and 8 weeks were involved in the 2-VO model. Although the level of PI3K protein in the 2-VO 1 week group was lower than that in the sham-operated group, it is persistently increased in the 2-VO 4 weeks rats and 2-VO 8 weeks rats. After 2-VO treatment, p-Akt protein was significantly increased in all 2-VO groups as compared to the sham-operated group. This study also showed a linear relationship between the PI3K protein expression and the behavioral measures of the cognitive abilities (i.e., the discrimination ratio and the time spent in the target quadrant NE).

Taken together, this study confirms the PI3K/Akt signal pathway may be involved in both the non-spatial and spatial cognitive impairment in CCH caused by the 2-VO model. There are several limitations to this study. First, we only observed the behavioral changes and the expression of PI3K/Akt protein in the hippocampus at three time intervals. The role of the PI3K/Akt signal pathway at other time points and other brain regions is not known. Second, we never used PI3K/Akt inhibitors or activators in an attempt to alter the behavior or expression of PI3K/Akt. Third, we did notexamine the relationships between neuron necrosis, apoptosis and their related genes with the behavioral changes and the expression of the PI3K/Akt protein. Further study is warranted to explore all of these questions, which will be essential in our understanding of the mechanisms of CCH and drug therapies that improve the cognitive impairment caused by cerebral hypoperfusion.
